# Supplementary material for: Climate Variables Related to the Incidence of Human Leishmaniosis in Montenegro in Southeastern Europe during Seven Decades (1945–2014)
Source: Int J Environ Res Public Health. 2023 Jan 17;20(3):1656. doi: 10.3390/ijerph20031656 (PMC9914530; doi:10.3390/ijerph20031656)
Supplement: Supplementary file 1 [file ijerph-20-01656-s001.zip › ijerph-2114225-supplementary.pdf]

Table S1. Year incident data reported for Leishmaniosis from 1945 - 2014

Year	Costal		Central		Highland		Total		Year	Costal		Central		Highland		Total	
	No.	Incidence	No.	Incidence	No.	Incidence	No.	Incidence		No.	Incidence	No.	Incidence	No.	Incidence	No.	Incidence
**1945**	0	0.00	0	0.00	0	0.00	0	0.00	**1980**	2	2.07	0	0.00	0	0.00	2	0.38
**1946**	0	0.00	0	0.00	0	0.00	0	0.00	**1981**	0	0.00	0	0.00	0	0.00	0	0.00
**1947**	0	0.00	0	0.00	0	0.00	0	0.00	**1982**	0	0.00	0	0.00	0	0.00	0	0.00
**1948**	5	7.16	3	2.33	0	0.00	8	2.12	**1983**	1	0.86	1	0.42	0	0.00	2	0.34
**1949**	2	2.87	6	4.66	0	0.00	8	2.12	**1984**	1	0.86	1	0.42	0	0.00	2	0.34
**1950**	2	2.87	1	0.78	0	0.00	3	0.80	**1985**	1	0.86	1	0.42	0	0.00	2	0.34
**1951**	1	1.43	2	1.55	0	0.00	3	0.80	**1986**	1	0.86	0	0.00	0	0.00	1	0.17
**1952**	4	5.73	3	2.33	0	0.00	7	1.86	**1987**	2	1.73	0	0.00	0	0.00	2	0.34
**1953**	1	1.31	1	0.69	0	0.00	2	0.48	**1988**	0	0.00	0	0.00	0	0.00	0	0.00
**1954**	1	1.31	3	2.07	0	0.00	4	0.95	**1989**	2	1.73	0	0.00	0	0.00	2	0.34
**1955**	0	0.00	0	0.00	0	0.00	0	0.00	**1990**	0	0.00	0	0.00	0	0.00	0	0.00
**1956**	0	0.00	3	2.07	0	0.00	3	0.71	**1991**	0	0.00	0	0.00	0	0.00	0	0.00
**1957**	1	1.31	0	0.00	0	0.00	1	0.24	**1992**	2	1.48	1	0.38	0	0.00	3	0.49
**1958**	0	0.00	4	2.76	2	1.01	6	1.43	**1993**	0	0.00	2	0.76	0	0.00	2	0.33
**1959**	5	6.57	2	1.38	1	0.50	8	1.91	**1994**	1	0.74	0	0.00	0	0.00	1	0.16
**1960**	3	3.94	1	0.69	0	0.00	4	0.95	**1995**	1	0.74	1	0.38	0	0.00	2	0.33
**1961**	1	1.20	0	0.00	0	0.00	1	0.21	**1996**	0	0.00	0	0.00	0	0.00	0	0.00
**1962**	1	1.20	0	0.00	0	0.00	1	0.21	**1997**	0	0.00	2	0.76	0	0.00	2	0.33
**1963**	0	0.00	0	0.00	0	0.00	0	0.00	**1998**	0	0.00	1	0.38	0	0.00	1	0.16
**1964**	0	0.00	2	1.17	0	0.00	2	0.42	**1999**	1	0.74	0	0.00	0	0.00	1	0.16
**1965**	1	1.20	2	1.17	0	0.00	3	0.64	**2000**	0	0.00	1	0.38	1	0.46	2	0.33
**1966**	1	1.20	1	0.59	0	0.00	2	0.42	**2001**	5	3.71	1	0.38	0	0.00	6	0.98
**1967**	5	5.99	1	0.59	0	0.00	6	1.27	**2002**	2	1.48	5	1.91	0	0.00	7	1.14
**1968**	1	1.20	1	0.59	0	0.00	2	0.42	**2003**	4	2.74	5	1.91	0	0.00	9	1.45
**1969**	0	0.00	0	0.00	0	0.00	0	0.00	**2004**	5	3.43	2	0.72	0	0.00	7	1.13
**1970**	0	0.00	0	0.00	0	0.00	0	0.00	**2005**	2	1.37	0	0.00	0	0.00	2	0.32
**1971**	0	0.00	2	0.99	0	0.00	2	0.38	**2006**	1	0.69	3	1.07	0	0.00	4	0.65
**1972**	0	0.00	0	0.00	0	0.00	0	0.00	**2007**	0	0.00	1	0.36	0	0.00	1	0.16
**1973**	1	1.03	0	0.00	0	0.00	1	0.19	**2008**	1	0.69	2	0.72	0	0.00	3	0.48
**1974**	2	2.07	1	0.49	0	0.00	3	0.57	**2009**	1	0.69	1	0.36	0	0.00	2	0.32
**1975**	2	2.07	0	0.00	0	0.00	2	0.38	**2010**	0	0.00	1	0.36	0	0.00	1	0.16
**1976**	0	0.00	0	0.00	0	0.00	0	0.00	**2011**	2	1.35	2	0.68	0	0.00	4	0.65
**1977**	0	0.00	1	0.49	0	0.00	1	0.19	**2012**	0	0.00	1	0.34	1	0.56	2	0.32
**1978**	0	0.00	0	0.00	0	0.00	0	0.00	**2013**	3	2.02	1	0.34	0	0.00	4	0.65
**1979**	0	0.00	0	0.00	0	0.00	0	0.00	**2014**	2	1.35	0	0.00	1	0.56	3	0.48
